# Influence of flow rate and fiber tension on dynamical, mechanical and acoustical parameters in a synthetic larynx model with integrated fibers

**DOI:** 10.3389/fphys.2024.1455360

**Published:** 2024-11-19

**Authors:** Lucia Gühring, Bogac Tur, Marion Semmler, Anne Schützenberger, Stefan Kniesburges

**Affiliations:** Department of Otorhinolaryngology, Medical School, Division of Phoniatrics and Pediatric Audiology, Friedrich-Alexander-Universität Erlangen-Nürnberg (FAU), Head and Neck Surgery, University Hospital Erlangen, Erlangen, Waldstrasse, Germany

**Keywords:** biomimetic larynx model, flow-induced vocal folds’ oscillations, fluid-structure-acoustic-interaction, physiological phonation characteristics, integrated fibers in synthetic larynx model

## Abstract

**Introduction:**

The human voice is generated by the oscillation of the vocal folds induced by exhalation airflow. Consequently, the characteristics of these oscillations and the primary sound signal are controlled by the longitudinal tension of the vocal folds, the flow rate, and their prephonatoric position. To facilitate independent control of these parameters, a synthetic larynx model was developed, as detailed in a previous publication.

**Methods:**

This study aims to statistically analyze the influence of airflow and fiber tension on phonation characteristics, such as periodicity and symmetry, glottis closure during vocal fold oscillations, as well as tissue elasticity and generated sound. A total of 76 experiments were conducted and statistically analyzed with a systematic variation of flow rate and longitudinal tension within the vocal folds.During these experiments, vocal fold motion, subglottal pressure, and emitted sound were meticulously measured and analyzed.

**Results:**

Groupwise statistical testing identified the flow rate as the main influencing parameter on nearly all phonation characteristics. However, the fundamental frequency, stiffness parameters, and quality parameters of the primary sound signal are predominantly controlled by the longitudinal tension within the vocal folds.

**Discussion:**

The results demonstrated a complex interplay between the flow rate and tension, resulting in different characteristics of the produced sound signal.

## 1 Introduction

The human voice plays a crucial role in social communication (Tiwari and Tiwari, 2012; Stewart et al., 2020; Kraus, 2017) and mental health (Bashshur and Oc, 2015; Rubin et al., 2019; Fagherazzi et al., 2021). In particular, the study of the physiology of the larynx, vocal folds, and acoustic voice production has made considerable progress in recent decades to gain a deeper understanding of this complex process and to develop new approaches for the diagnosis and therapy of vocal fold pathologies, such as vocal atrophy (Nguyen et al., 2009; Choi et al., 2012; Morrison et al., 1983; Van Den and Tan, 1959; Martins et al., 2015). The primary acoustic voice signal is generated and controlled deep in the larynx due to the fluid-structure interaction between the exhalation airflow from the lungs and the hyperelastic vocal folds (Mohri et al., 1998; Iwarsson et al., 1998).

Due to the significant ethical and practical limitations associated with experimental analysis of the phonation process in living humans or animals, synthetic larynx models have been developed to serve as a controlled and reproducible test environment. These models enable the investigation of all aspects of the biomechanical phonation process, as they are scientifically valid (Scherer et al., 2001; Barney et al., 1999; Deverge et al., 2003; Cisonni et al., 2008; Kniesburges et al., 2020) classified vocal-fold models into three main groups according to their experimental strategy: static, driven, and fully coupled models. By integrating fibers into the model, the biomechanical properties of the human larynx can be simulated, allowing for a more comprehensive exploration of the interactions among airflow, tension, and the resulting response during phonation (Vahabzadeh‐Hagh et al., 2018; Xuan and Zhang, 2014; Shaw et al., 2012; Murray and Thomson, 2012). In that context, this study focused on the statistical evaluation of data measured from a synthetic silicone laryngeal model with integrated fibers, as described by Tur et al. (2023). This model not only includes fibers in the ligament layers but can also reproduce laryngeal movements, such as adduction and elongation.

This study aimed to investigate the complex relationships between airflow, prephonatory tension, and motion of the vocal folds within the synthetic larynx based on glottal and acoustic parameters. Statistical analysis was performed to identify the effects of flow rate and tension on periodicity, symmetry, and glottis closure during oscillation, as well as tissue elasticity and produced sound.

## 2 Methods

### 2.1 The synthetic larynx model

**FIGURE 1:**
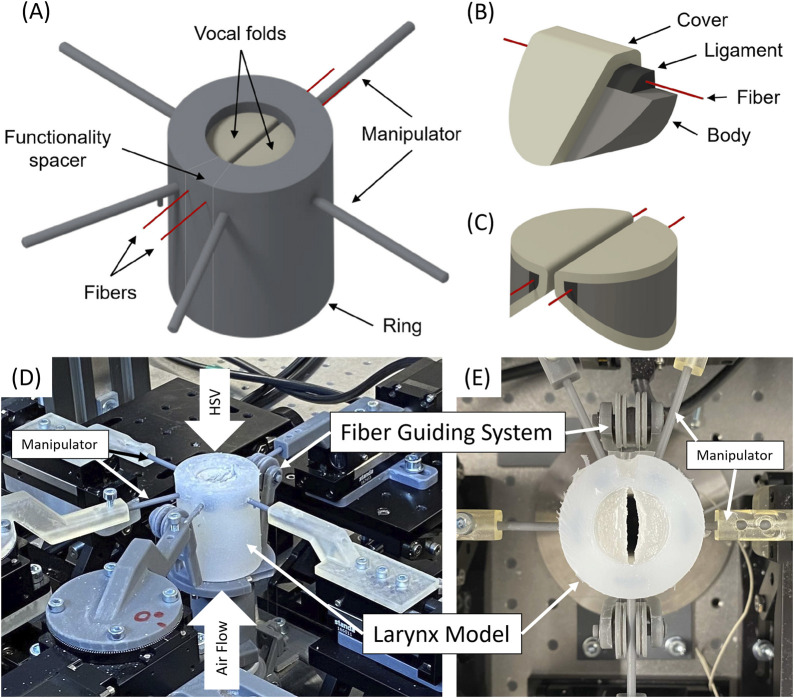
**(A)** 3D CAD model of the entire larynx, including the manipulators, the vocal folds (based on the M5 model), the ring, and the fibers **(B)** Detailed view of the individual layers in the vocal fold model, showing the body, ligament, fiber, and cover. **(C)** 3D perspective of the vocal folds arranged within the larynx model. **(D)** Perspective view of the measurement setup with the mounted model showing air flow supply, highspeed video (HSV) camera positioning. **(E)** Top view of the model showing the fiber guiding system.

The artificial larynx model introduced by Tur et al. (2023) was investigated concerning the impact of the flow rate and fiber tension in integrated fibers on different dynamic, acoustic, and mechanical parameters related to voice production. The exact geometry, fabrication procedure, and material composition of the model are presented in Figure 1.

The synthetic vocal folds possess the M5 geometry described by Scherer et al. (Barney et al., 1999) and consist of three silicone layers that reproduce the anatomical structures of human vocal folds, including the vocalis muscle, ligament, and lamina propria (Scherer et al., 2001). Additionally, fibers have been integrated into the ligament to vary the longitudinal tension in vocal fold models. In the experiment, the fibers were stretched to a certain length without explicitly measuring the tension.

Synthetic vocal folds were embedded within a silicone cylinder, which additionally included manipulators to control the prephonatory position of the vocal folds.

The integrated fibers had a diameter of 0.108 mm and a breaking resistance of 1.18 kg. They consisted of polyvinylidene fluoride (PVDF) with the following dynamic mechanical properties: E′-modulus of 2.91 GPa (0.07 standard deviation), E´´-modulus of 0.08 GPa (0.01 GPa standard deviation) and loss-tangent tanδ of 0.03 (0.00 standard deviation). Detailed information regarding the manufacturing process can be found in Tur et al. (2023).

### 2.2 Measurement setup

To control longitudinal tension in the ligament fibers, a 7T67-25 Stab. A Steel Translation Stage (Standa Ltd., Vilnius, Lithuania) with a linear travel range of 25 mm is used. To reduce friction, a customized guiding system was developed to direct the fibers parallelly through the two vocal folds.

The flow rate was controlled using a 1578 A/B mass flow controller (MKS, Andover, MA, United States) and a 4000 B 154 digital power supply (MKS) (Birk et al., 2017a; Semmler et al., 2021; Jakubaß et al., 2023; Peters et al., 2022). The flow rate amounted to 30–200 standard liters per minute (SLM), with the lower value being the flow rate for oscillation onset. Subglottal pressure was measured using an XCS-93-5PSISG pressure sensor (Kulite Semiconductor Products, Inc., Leonia, NJ, United States) positioned approximately 130 mm below the glottal plane connected to a PXle-4330 bridge module (National Instruments, Austin, TX, United States) with a sampling frequency of 44.1 kHz.

High-speed videos of glottal dynamics were captured using a Phantom V2511 high-speed digital camera (Vision Research, Wayne, NJ), United States) at a frame rate of 4,000 frames/s (fps) with an image resolution of 768 × 768 pixels and a recording time of a minimum of 600 ms. A Canon EF 180 mm f/3.5 L macro lens (Canon Inc., Tokyo, Japan) projected the vocal folds on a camera chip.

Acoustic signals were recorded in the supraglottal region using two 4,189 1/2-inch free-field microphones (Brüel and Kjær, Nærum, Denmark) at 30 cm from the model and sampled with a frequency of 44.1 kHz for a duration of 1s using a PXLe-4492 sound and vibration module (National Instruments, Austin, TX).

Acoustic and pressure data were acquired simultaneously using a LabVIEW script.

The model was mounted on an artificial trachea, and the fibers were secured to a linear translation stage. The translation stage, with a range of 0–25 mm, was initially set at 5 mm to represent minimal tension.

It was ensured that the fibers were in a completely tension-free state by setting the translation stage to 0 mm. The vocal folds were then adducted to achieve complete glottal closure. The flow rate was gradually increased until the synthetic vocal folds began to oscillate stably, at which point measurement was started. The flow rate was then increased in increments of 10 SLM, taking measurements at each step until reaching the maximum flow rate of 200 SLM.

After completing these measurements on one fiber tensioning level, the flow was turned off, and the fiber tension was increased by elongating the fibers by 5 mm. The glottal closure was reestablished if necessary, and the measurement process was repeated starting from the onset flow level of oscillation. This protocol was repeated at six different tension levels, with the maximum fiber elongation set at 20 mm, representing the highest tension level. This resulted in a total of N = 76 measurements for the flow ranges between 30 SLM and 200 SLM for fiber tensioning levels from 0 mm to 25 mm (Tur et al., 2023).

### 2.3 Data acquisition

Table 1 shows 13 key phonation parameters (glottal dynamic, mechanical, acoustic) evaluated within this study. Additionally, the fundamental frequency and subglottal pressure are considered to represent general phonation parameters (Semmler et al., 2021).

1. Subglottal Pressure

**TABLE 1:** Overview of all considered parameters in this publication. The term “higher/smaller is better” refers to physiological modal phonation regarding efficiency, regularity, harmony, and noise (Titze and for, 1995).

Parameter	Debriviation (Unit)	Description	Range/Categorization	References
General Phonation Parameter				
Fundamental Frequency	F0 (Hz)	Frequency at which oscillates	Physiological range: 100–220 Hz	Falk et al. (2021)
Subglottal Pressure	Psub (Pa)	Pressure measured beneath vocal folds	Physiological range: 160–3,510 Pa	Falk et al. (2021)
Periodicity and Symmetry				
Amplitude Periodicity	AP (AU)	Cycle-based periodicity regarding amplitude	1 = periodic [0; 1]	Qiu et al. (2003)
Time Periodicity	TP (AU)	Cycle-based periodicity regarding time	1 = periodic [0; 1]	Qiu et al. (2003)
Amplitude Symmetry Index	ASI (AU)	Size difference of maximal left to right glottal area	1 = symmetric [0; 1]	Wang et al. (2016)
Phase Asymmetry Index	PAI (AU)	Phase difference in left and right glottal area dynamics	0 = symmetric [0; 1]	Qiu et al. (2003), Patel et al. (2014a)
Glottis Closure				
Glottis Gap Index	GGI (AU)	Ratio of minimum and maximum glottal opening	1 = completely open [0; 1]	Patel et al. (2014a)
Closing Quotient	ClQ (AU)	Quotient of time with full glottal closure	0 = full closure [0; 1]	Holmberg et al. (1988)
Maximum Area of Declination	MADR (Mpx/s)	Maximum glottal closing speed regarding vocal fold fiber tension	Higher is faster	Schlegel et al. (2019)
Tissue Elasticity				
Stiffness	stiff (1/s)	Glottal closing and opening speeds regarding the tissue	Higher is stiffer Higher is faster	Munhall et al. (1985), Titze and Martin (1998), Stevens (2000)
Amplitude-to-Length-Ratio	ALR (AU)	Glottal elongation during oscillation	Lower is stiffer	Schlegel et al. (2019)
Acoustics				
Cepstral Peak Prominence	CPP-II (dB)	Cepstral measure of acoustic voice quality	Higher is better	Hillenbrand et al. (1994), Pelka et al. (2023)
Harmonics-to-Noise-Ratio	HNR (dB)	Shows the extent to which harmonic structure is overlapped by noise	Higher is better	Yumoto et al. (1982)
Jitter	jitt (%)	Average variation in the durations of two succeeding cycles	Smaller is better	Bielamowicz et al. (1996)
Shimmer	shim (%)	Normalized average in amplitude between two cycles	Smaller is better	Titze et al. (1987)

Based on the subglottal pressure signal, the mean subglottal pressure and fundamental frequency of vocal-fold oscillation were computed using MATLAB R2021b (The MathWorks, Inc., Natick, MA, United States).

2. GAW Dynamic and Mechanical Parameters

For the analysis of vocal fold dynamics, glottal segmentation, and subsequent analysis were performed using the in-house program Glottis-Analysis-Tool 2020 (GAT) (Maryn et al., 2020; Kist et al., 2021) by extracting the glottal area waveform (GAW), which is the glottis area as a function of time. A minimum of 20 consecutive cycles were used to ensure stable results (Schlegel et al., 2018; Karnell et al., 1995). Based on the GAW, parameters that can be divided into three categories were computed:

- Periodicity and Symmetry

Amplitude Periodicity (AP), Time Periodicity (TP), Amplitude Symmetry Index (ASI) and Phase Asymmetry Index (PAI).

- Glottis closure characteristics:

Parameters: Glottis Gap Index (GGI) Closing Quotient (ClQ) and maximum area of decline (MADR).

- Tissue elasticity:

Parameters: Stiffness (stiff) and Amplitude-to-Length-Ratio (ALR).

The stiffness parameter as a measure of tissue elasticity is a pure quantity derived from motion characteristics of the vocal folds recorded by the high speed camera, see Equation 1 (Munhall et al., 1985)

$$stiff=\frac{\max_{t\subset T_{i}}\left(s\left(t\right)\right)}{A_{i}}$$ (1)

The *stiff* quantifies the maximum closing and opening speed of the glottis relative to the dynamic range A_i_ of the glottal area waveform (GAW) for the *i*th cycle. Here, s(t) represents the absolute magnitude of the first derivative of the GAW for the *i*th cycle, with T_i_ being the duration of that cycle (Munhall et al., 1985). A lower *stiff* value indicates a slower glottal opening and closing speed. Generally, this parameter is interpreted as follows: a higher s*tiff* value suggests increased tissue stiffness, as the vocal folds move faster under tension, thereby reducing their flexibility during rapid movement (Kist et al., 2021; Pelka et al., 2023; Titze and Martin, 1998; Roy, 2003).

3. Acoustic Parameters

The acoustic signals were analyzed for 100 oscillation cycles within the GAT (Alipour et al., 1997; Mehta et al., 2011). 2 parameter categories were focused on:

- Sound quality measures:

Parameters: Cepstral Peak Prominence (CPP) and Harmonics-to-Noise-Ratio (HNR).

- Regularity:

Parameters: Jitter (jitt) and Shimmer (shim).

### 2.4 Statistical data analysis

The statistical analysis was carried out with respect to the following questions:

1. What influence does the flow rate have on the parameters?

To analyze the impact of the flow rate, the experimental dataset was divided into four flow groups, as shown in Table 2. FG1 and FG2 represent the physiological range of flow rates typically observed during exhalation ranging from 48 to 112 L/min (Scheinherr et al., 2015), while FG3 and FG4 correspond to non-physiological conditions. These non-physiological flow ranges were still considered, as they potentially show important relationships. The flow groups contain a similar number of measurements, ensuring that the statistical analysis is not biased by unequal sample sizes. N indicates the number of data points within each respective flow rate range.

2. What influence does the fiber tension have on the parameters?

**TABLE 2:** Overview of all flow rate groups with their corresponding values.

Group	Flow rate (SLM)	N
FG1	30, 32, 34, 36, 38, 40	18
FG2	50, 60, 70, 80	18
FG3	90, 100, 110, 120	20
FG4	130, 140, 150, 160, 170, 180, 190, 200	20

To determine the influence of longitudinal fiber tension, five different stretching lengths (SL) were compared, indicating fiber tension using a pairwise test. Fiber elongation was performed in the total range of 0 mm–20 mm in 5 mm increments. This resulted in the pairwise comparisons presented in Table 3.

**TABLE 3:** Overview of the comparison tests regarding the fiber tension. The fiber tension level is indicated by the stretching lengths.

Pairwise test	Stretching length (SL)
S1	0 mm–5 mm
S2	0 mm–10 mm
S3	0 mm–15 mm
S4	0 mm–20 mm
S5	5 mm–10 mm
S6	5 mm–15 mm
S7	5 mm–20 mm
S8	10 mm–15 mm
S9	10 mm–20 mm
S10	15 mm–20 mm

All statistical analyses were performed using SPSS version 29 (IBM Corp., Armonk, NY, United States). Kruskal-Wallis tests were performed at a significance level of *p* < 0.05. Nonparametric Mann-Whitney-U-Tests were applied *post hoc* for pairwise comparisons. According to the Bonferroni correction, the significance level was calculated as *p* < 0.0083 for the flow rate groups FG1-FG4 and *p* < 0.005 for the comparison of stretching length S1-S10.

## 3 Results

### 3.1 Global statistical overview

The results of the statistical analysis are presented in combination with classical boxplots, which show the distribution of measurements for each group. The horizontal line within the box represents the median value across all measurements in the group, while the cross indicates the mean value. Outliers are additionally depicted by circles.

#### 3.1.1 Flow rate impact

**FIGURE 2:**
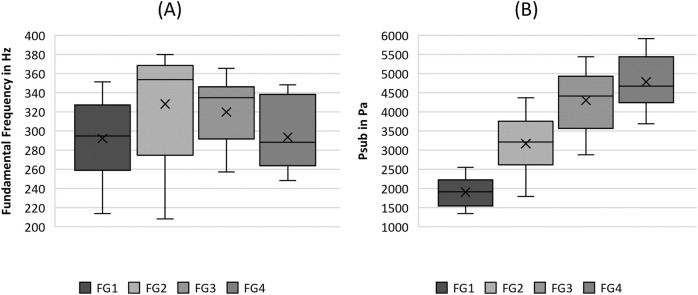
Boxplot representation of the **(A)** fundamental frequency and the **(B)** subglottal pressure as function of flow rate. The horizontal line within the box corresponds to the median, the cross to the mean value. Outliers are indicated by circles.

**FIGURE 3:**
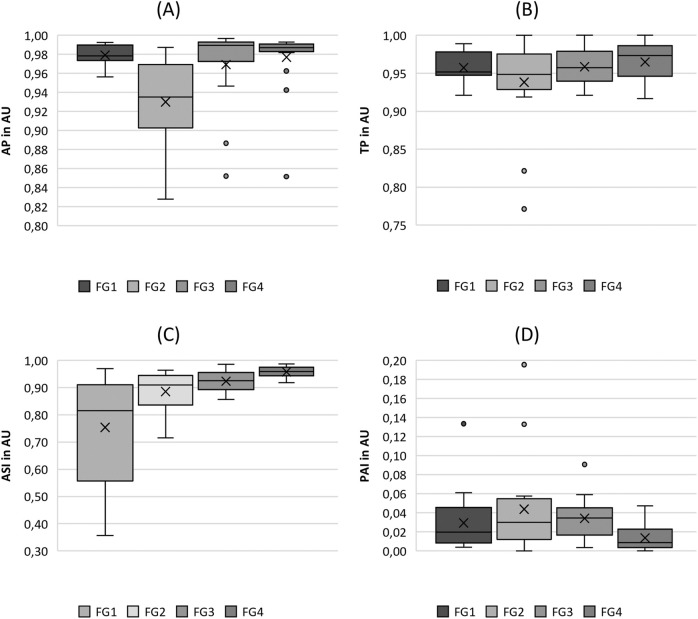
Boxplot representation of the **(A)** amplitude periodicity (AP), **(B)** time periodicity (TP), **(C)** amplitude symmetry index (ASI) and **(D)** phase asymmetry index (PAI) as function of flow rate. The horizontal line within the box corresponds to the median, the cross to the mean value. Outliers are indicated by circles.

**FIGURE 4:**
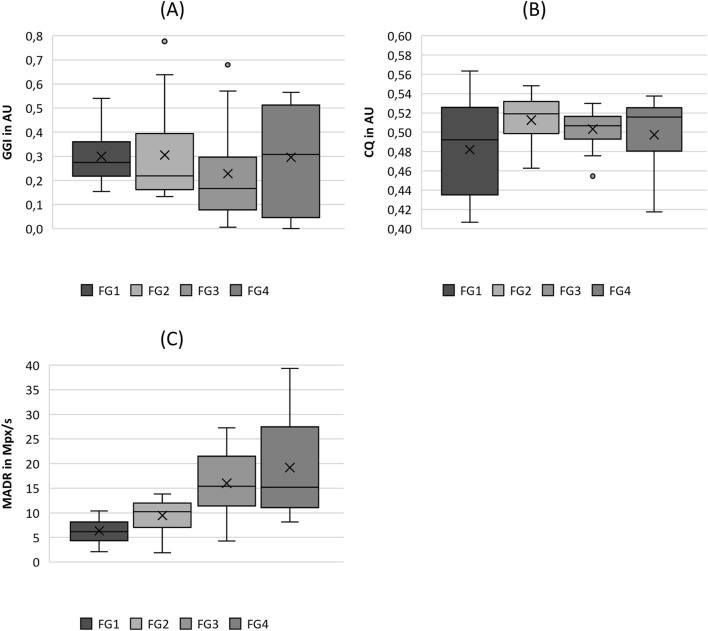
Boxplot representation of the **(A)** glottis gap index (GGI), **(B)** closing quotient (CIQ) and **(C)** maximum area of declination (MADR) as function of flow rate. The horizontal line within the box corresponds to the median, the cross to the mean value. Outliers are indicated by circles.

**FIGURE 5:**
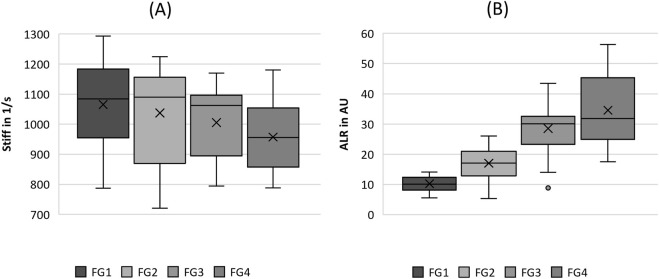
Boxplot representation of the **(A)** stiffness (stiff) and **(B)** amplitude-to-length-ratio (ALR) as function of flow rate. The horizontal line within the box corresponds to the median, the cross to the mean value. Outliers are indicated by circles.

**FIGURE 6:**
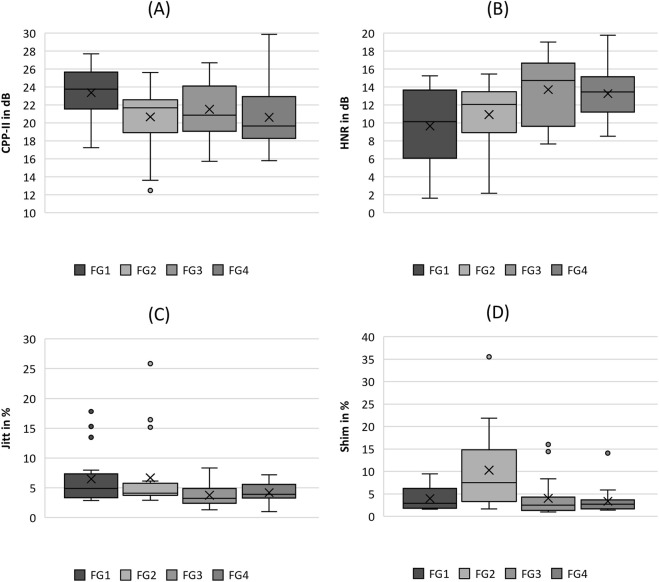
Boxplot representation of the **(A)** cepstral peak prominence (CPP), **(B)** harmonics-to-noise-ratio (HNR), **(C)** jitter (jitt) and **(D)** shimmer (shim) as function of flow rate. The horizontal line within the box corresponds to the median, the cross to the mean value. Outliers are indicated by circles.

The statistical analysis, in combination with Figures 2–6 indicates that the flow rate influences nearly all parameter groups, most prominently the General Phonation Parameters, Symmetry, and Acoustics groups, as listed in Table 4.

**TABLE 4:** Overview of the Kruskal-Wallis test and *post hoc* for the influence of flow rate on all parameters.

Influence of flow rate							
	Kruskal-Wallis (*p* < 0.05)	Post hoc (Mann-Whitney-U; *p* < 0.0083)					
		FG1-FG2	FG1-FG3	FG1-FG4	FG2-FG3	FG2-FG4	FG3-FG4
General Phonation Parameter							
F0	**0,009**	0.015	0.054	0.872	0.152	0.011	0.026
Psub	**<,001**	**<,001**	**<,001**	**<,001**	**<,001**	**<,001**	0.070
Periodicity and Symmetry							
AP	**<,001**	**<,001**	0.198	0.144	**0,001**	**<,001**	0.675
TP	0.355	-	-	-	-	-	-
ASI	**<,001**	0.018	**0,001**	**<,001**	0.114	**<,001**	**0,003**
PAI	**0,002**	0.242	0.279	0.024	0.861	**0,002**	**0,001**
Glottis Closure							
GGI	0.384	-	-	-	-	-	-
ClQ	0.233	-	-	-	-	-	-
MADR	**<,001**	**0,003**	**<,001**	**<,001**	**0,001**	**0,001**	0.607
Tissue Elasticity							
Stiff	0.102	-	-	-	-	-	-
ALR	**<,001**	**<,001**	**<,001**	**<,001**	**<,001**	**<,001**	0.160
Acoustics							
CPP	**0,020**	0.012	0.047	**0,008**	0.501	0.430	0.234
HNR	**0,005**	0.393	**0,004**	**0,007**	0.019	0.061	0.482
Jitt	**0,050**	0.849	0.014	0.198	0.028	0.242	0.224
Shim	**0,002**	**0,006**	0.254	0.413	**0,002**	**0,001**	0.534

Values printed in bold lettering indicate statistical significance with values lower than the p-value.

The *post hoc* tests indicated a decreasing influence with increasing flow rate, as there were only two parameters (ASI and PAI) that exhibited significant differences between flow rates FG3 and FG4.

Thus, changes in the flow rate from 90SLM SLM to 200SLM (FG3 and FG4) rarely generated significant differences in the parameters.

For all other pairwise comparisons, the proportions of significant differences in the parameters were approximately equally distributed.

#### 3.1.2 Fiber tension impact

The main influence of fiber tension was observed in F0, Stiffness, GGI, and CPP. One parameter group with no significant differences between the Periodicity and Symmetry of the fiber tension was found. Furthermore, it is noticeable that for S8, S9, and S10, there are no longer significant changes in any of the parameters, indicating that increasing the stretching length from 10 to 20 mm no longer affects the evaluated parameters.

### 3.2 Influence of flow rate on phonation parameters

#### 3.2.1 General phonation parameters

The analysis demonstrated lower F0 values for flow rates FG1 and FG4, whereas FG2 and FG3 show higher values of F0 as displayed in Figure 2A. Although F0 demonstrated significant differences in flow rate in Table 4, all pairwise comparisons of the single flow rate groups were not significant.

The P_sub_ was found to increase with increasing flow rate, as illustrated in Figure 2B. According to Table 4, this increase is indicated by the significance in the Kruskal-Wallis test as well as in most pairwise tests, except for the comparison between FG3 and FG4.

#### 3.2.2 periodicity and symmetry

Looking at the periodicity parameters in Figure 3, fairly constant values for both AP and TP were observed, ranging from 0.828 to 0.997 (mean: 0.964) for AP and from 0.771 to 1.000 (mean: 0.956) for TP, indicating a high level of periodicity. An exception was the flow rate group FG2 for the Amplitude Periodicity (AP), which demonstrated slightly lower values (mean: 0.93) than the other flow rates (0.975). Thus, the statistical tests yielded only statistical differences for comparisons with FG2, as listed in Table 4.

In Figure 3C, the ASI shows an increasing trend with increasing flow rate, indicating a larger symmetry of the vocal fold oscillations at larger flow rates. The statistical significance of the parameters also indicated this trend, although the pairwise tests between FG2, FG1, and FG3 were computed as insignificant (see Table 4). The PAI (Figure 3D) did not exhibit a clear trend. However, it indicated a decrease in asymmetry at higher flow rates, which was statistically significant for the comparison of FG2 with FG3 and FG4, as listed in Table 4.

#### 3.2.3 Closure

As depicted in Figure 4A, there is a slight tendency for the GGI to decline with increasing flow rate, with the exception of FG4, which exhibits a wider range including GGI = 0, representing complete glottis closure. However, this trend was not indicated by the significant differences between the flow rate groups, as listed in Table 4. The same holds for ClQ, which does not show any reasonable trend, as illustrated in Figure 4B. In contrast, as illustrated in Figure 4C, the MADR revealed a statistically significant difference in increasing values with increasing flow rate, with the only exception being the pairwise comparison between FG3 and FG4.

#### 3.2.4 Tissue elasticity

Regarding the tissue elasticity-related parameters in Figure 5, the stiffness seemed to decrease with increasing flow rate; however, no statistical difference between the flow rate groups was observed, as listed in Table 4. However, ALR exhibited a clear rising trend with increasing flow rate, as illustrated in Figure 5B. Similarly, for the MADR, the significant trend was only violated by the non-significant difference between FG3 and FG4, although the basic trend was still visible.

#### 3.2.5 Acoustics

In the acoustic group, the CPP and HNR parameters demonstrated a clear trend with increasing flow rate, as displayed in Figures 6A, B, with statistically significant differences according to the Kruskal-Wallis test, as listed in Table 4. The trends were opposite, that is CPP decreased and HNR increased with increasing flow rate. Evaluating the pairwise tests in Table 4, however, this relationship is weak because only the comparison between FG1, FG3, and FG4 was statistically significant in the pairwise tests.

In contrast, the two parameters Jitter and Shimmer do not show a reasonable trend for increasing flow rate, as illustrated in Figures 6C, D, although both demonstrated significant differences with increasing flow rate (see Table 4). Furthermore, the pairwise comparisons were not significant, except for Shimmer, which exhibited a larger range and median value for FG2, similar to the AP parameter from the periodicity group.

### 3.3 Influence of fiber tension indicated by the stretching length on phonation parameters

#### 3.3.1 General phonation parameters

**FIGURE 7:**
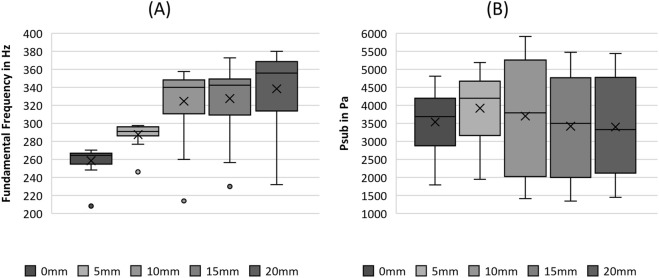
Boxplot representation of the **(A)** fundamental frequency and the **(B)** subglottal pressure as function of flow rate. The horizontal line within the box corresponds to the median, the cross to the mean value. Outliers are indicated by circles.

A clear rising trend in F0 can be observed with increasing stretching length (SL), as illustrated in Figure 7A. This trend holds up to a 10 mm elongation of the fibers. For higher stretching, F0 remained constant, which is also obvious in the pairwise tests demonstrating non-significant differences for the comparisons of SL of 10, 15, and 20 mm (S8-S10), as listed in Table 5.

**TABLE 5:** Overview of the Kruskal-Wallis test and *post hoc* for the influence of fiber tension on all parameters.

Influence of fiber tension											
	Kruskal-Wallis (*p* < 0.05)	Post hoc (Mann-Whitney-U; *p* < 0.005)									
		S1	S2	S3	S4	S5	S6	S7	S8	S9	S10
General Phonation Parameters											
F0	**<,001**	**<,001**	**<,001**	**<,001**	**<,001**	**0,002**	**0,002**	**0,002**	0.782	0.094	0.183
Psub	0.840	-	-	-	-	-	-	-	-	-	-
Periodicity and Symmetry											
AP	0.501	-	-	-	-	-	-	-	-	-	-
TP	0.096	-	-	-	-	-	-	-	-	-	-
ASI	0.054	-	-	-	-	-	-	-	-	-	-
PAI	0.074	-	-	-	-	-	-	-	-	-	-
Glottis Closure											
GGI	**<,001**	**<,001**	**<,001**	**<,001**	**<,001**	**0,007**	**0,002**	0.008	0.581	0.362	0.480
ClQ	0.298	-	-	-	-	-	-	-	-	-	-
MADR	**0,044**	**<,001**	0.055	0.030	0.123	0.968	0.503	0.155	0.783	0.494	0.561
Tissue Elasticity											
Stiff	**<,001**	**<,001**	**<,001**	**<,001**	**<,001**	**<,001**	**<,001**	**<,001**	0.535	0.095	0.096
ALR	0.114	-	-	-	-	-	-	-	-	-	-
Acoustics											
CPP	**<,001**	0.555	**<,001**	**<,001**	**<,001**	**<,001**	**0,001**	**<,001**	0.078	0.287	0.383
HNR	**0,001**	**<,001**	0.006	0.009	0,31	0.378	0.017	0.025	0.427	0.138	0.244
Jitt	**<,001**	**<,001**	**<,001**	**0,001**	0,02	0.968	0,11	0.105	0.168	0.102	0.561
Shim	0.051	-	-	-	-	-	-	-	-	-	-

Values printed in bold lettering indicate statistical significance with values lower than the p-value.

P_sub_ seemed to increase until a stretching length of 5 mm was reached, followed by an overall decrease, as depicted in Figure 7B. However, the statistical analysis revealed that P_sub_ displayed no significant differences between the different fiber tensions, as listed in Table 5.

#### 3.3.2 Periodicity and symmetry

**FIGURE 8:**
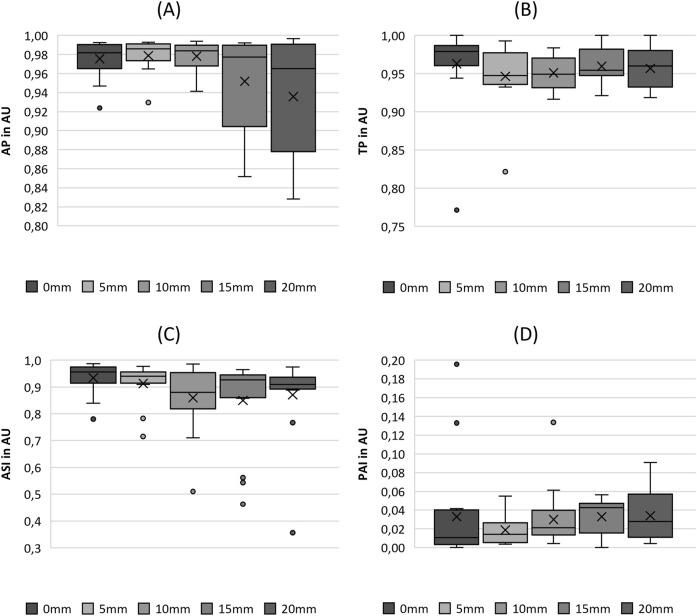
Boxplot representation of the **(A)** amplitude periodicity (AP), **(B)** time periodicity (TP), **(C)** amplitude symmetry index (ASI) and **(D)** phase asymmetry index (PAI) as function of fiber tension. The horizontal line within the box corresponds to the median, the cross to the mean value. Outliers are indicated by circles.

Both periodicity-related parameters, AP and TP, exhibit extremely high values, indicating the high periodicity of vocal fold oscillations, as displayed in Figures 8A, B. The apparent decreasing trend with increasing fiber stretching length was not statistically significant owing to the nonsignificant occurrences, as listed in Table 5. Interestingly, the range of values for the AP explicitly increased for the two largest stretching levels: 15 and 20 mm.

Similar to the Periodicity parameters, the ASI and PAI parameters demonstrate extremely high and low values, respectively, as depicted in Figures 8C, D. They indicated highly symmetric motion of the two vocal folds. Accordingly, the statistical tests demonstrated no significant differences between the stretching levels and symmetry, as listed in Table 5.

#### 3.3.3 Closure

**FIGURE 9:**
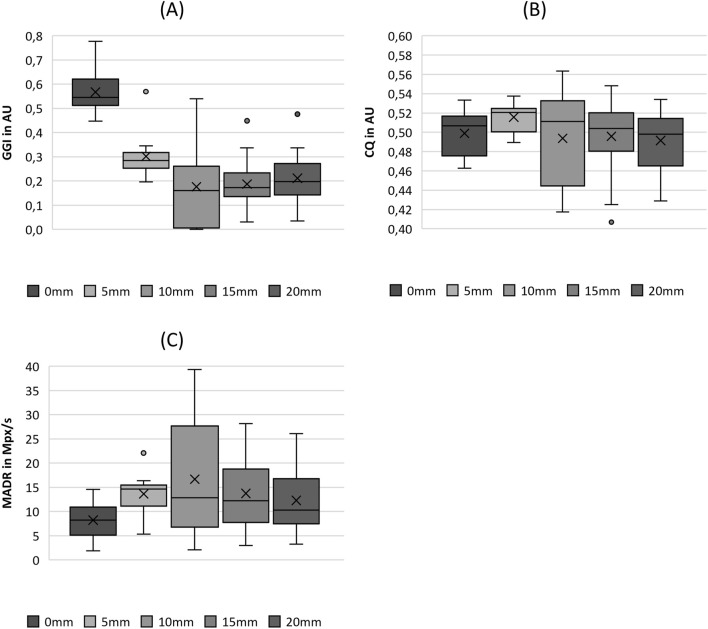
Boxplot representation of the **(A)** glottis gap index (GGI), **(B)** closing quotient (CIQ) and **(C)** maximum area of declination (MADR) as function of fiber tension. The horizontal line within the box corresponds to the median, the cross to the mean value. Outliers are indicated by circles.

The GGI shows a range between 0.00 and 0.78 with the highest value (0.78) at SL 0 mm and the lowest value (0.00) at SL 10 mm. It decreased with increasing fiber stretching until a value of 10 mm was reached. For larger stretching, the GGI remained fairly constant, as depicted in Figure 9A, with no statistical differences between the fiber stretching levels, as illustrated by the pairwise test in Table 5.

The Closing Quotient showed no systematic trend, as indicated by the insignificant Kruskal-Wallis test in Table 5.

Although MADR showed statistically significant differences in the fiber stretching length, the distribution in Figure 9C was similar to that of ClQ in Figure 9B, with an initial increase in the median values followed by a decrease.

#### 3.3.4 Tissue elasticity

**FIGURE 10:**
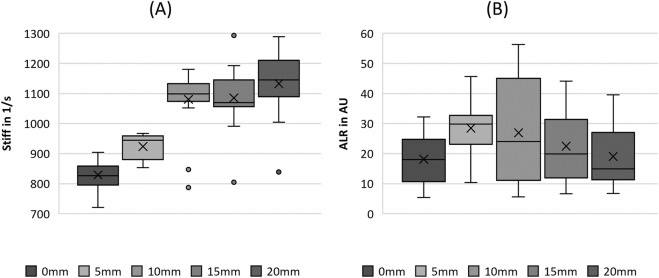
Boxplot representation of the **(A)** stiffness (stiff) and **(B)** amplitude-to-length-ratio (ALR) as function of fiber tension. The horizontal line within the box corresponds to the median, the cross to the mean value. Outliers are indicated by circles.

The increase in fiber tension, as indicated by the stretching length, increased the stiffness up to an SL of 15 mm, as illustrated in Figure 10A. For larger stretching levels, the stiffness did not change significantly, as revealed by the pairwise tests in Table 5. The ALR displayed in Figure 10B illustrates a similar non-significant variation, with an initial increase in the median up to an SL of 10 mm, followed by a decrease for further fiber elongation, as reported previously for parameters such as MADR, ClQ, and P_sub_.

#### 3.3.5 Acoustics

**FIGURE 11:**
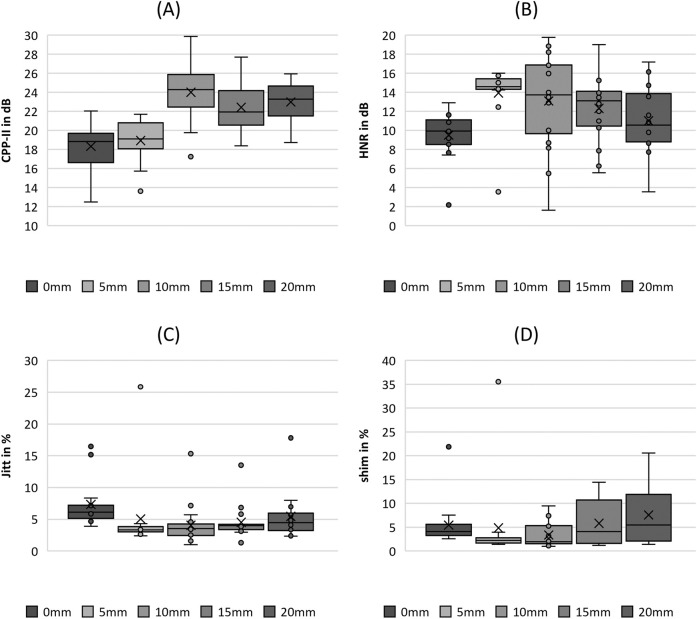
Boxplot representation of the **(A)** cepstral peak prominence (CPP), **(B)** harmonics-to-noise-ratio (HNR), **(C)** jitter (jitt) and **(D)** shimmer (shim) as function of fiber tension. The horizontal line within the box corresponds to the median, the cross to the mean value. Outliers are indicated by circles.

CPP exhibited a trend of increasing with increasing fiber stretching length up to an SL of 10 mm and followed by a decrease in values with further stretching, as depicted in Figure 11A. Therefore, the increase showed statistically significant differences, as shown in the pairwise *post hoc* tests in Table 5. Only the change in the values from 0 mm to 5 mm was not significant. A further decrease in CPP was not analogous to the parameters Stiff, GGI, and F0.

The HNR also showed a tendency to increase with increasing fiber stretching until it reached a value of 10 mm, as depicted in Figure 11B. This increase is indicated as well by statistical significance, as listed in Table 5. A further decrease in the HNR was not significant.

The values for jitter decrease in Figure 11C as the fiber stretching length increases up to an SL of 10 mm, which is statistically significant. In contrast, Shimmer did not show statistical significance with increasing fiber stretching levels. However, as illustrated in Figure 10D, the range increases for stretching levels up to 20 mm.

## 4 Discussion

### 4.1 Impact of flow rate on phonation parameters

Considering the flow rate, it was found that its influence could be clearly observed in all six parameter groups, as listed in Table 4. However, there were groups in which the influence of the flow rate was greater than that in other groups.

The flow rate has a significant influence on the P_sub_. According to physiological conditions, as well as to previous measurements on synthetic larynx models and excised larynges described in previous studies, the P_sub_ is linearly related to the airflow (Semmler et al., 2021; Alipour et al., 1997; Döllinger et al., 2012; Birk et al., 2017b). This linearity can be found in the data and agrees with the findings of the statistical analysis performed, proving the flow-rate controlled model to be oscillating in a physiological manner. Although the values of P_sub_ are higher than the physiological values in the range of approximately 350–3,510 Pa (Falk et al., 2021; Holmberg et al., 1988; Sundberg et al., 1993; Sundberg et al., 2005; Baken, 1987), the trend is still similar to an P_sub_ increase with an increasing flow rate (Alipour et al., 1997; Sundberg et al., 1993; Zhang, 2015). Thus, the larger the flow rate, the higher is P_sub_ (Chhetri and Park, 2016; Guzman et al., 2019). In contrast, the fundamental frequency demonstrated no trend that would be systematically related to the flow rate, as statistical tests revealed (Semmler et al., 2021; Tong and Sataloff, 2022; Asnaashari et al., 2012). The trend demonstrated a short increase in the F0 median and range, followed by a decrease.

Regarding the oscillation characteristics of the vocal folds, a significant influence of the flow rate only on the AP can be observed. However, this was the result of an increase in the range of AP, with a decrease in the median only for the second flow rate group FG2. This mechanism can also be observed in the Shimmer for the acoustic signal, which shows a strong relationship between the oscillation pattern of the vocal folds and the resulting sound production (Farrús et al., 2007). It can be suspected that FG2 has a certain flow rate range that leads to instability in the oscillations of the amplitude sequences.

For the other flow rate groups, there were fairly constant high values for AP, indicating oscillation characteristics similar to those of regularly oscillating human vocal folds (Jakubaß et al., 2023; Peters et al., 2022). The same trend was observed for TP.

Vocal fold oscillations demonstrated a high level of symmetry, as both the ASI and PAI lie in the physiological range of regular human vocal fold oscillations (Schlegel et al., 2020; Bonilha et al., 2012; Schützenberger et al., 2016). Based on the statistically significant relationship between symmetry parameters and flow rate, the model reproduces the stabilizing effect of increasing the flow rate to maintain symmetrical oscillations of the vocal folds (Semmler et al., 2021).

The oscillation of the vocal folds showed a pattern with and without glottic closure, as shown by the GGI. Therefore, glottis closure was only achieved for higher flow rates that produced larger oscillation amplitudes (Semmler et al., 2021). ClQ has a physiological value (Semmler et al., 2023; Verdolini et al., 1998; Patel et al., 2014a). However, a strong relationship between flow rate and glottic closure could not be found in the model. This agrees with recent studies, where Taylor et al. have shown through a synthetic vocal fold model, that glottis closure is in fact mainly controlled by the vocal fold geometry, such as thickness, rather than flow rate or fiber tension (Taylor and Thomson, 2022).

In contrast, the MADR increased with increasing flow rate, which has also been reported previously (Laukkanen et al., 2008; Titze and Palaparthi, 2016). As the MADR has been reported to correlate with the strength of discrete acoustic tones produced by the oscillations of the vocal folds (Titze, 2006; Kniesburges et al., 2020), the harmonic-to-noise ratio (HNR) of the acoustic signal also increases with the flow rate. Simultaneously, the CPP demonstrated a slight decrease with increasing flow rate, which seems to contradict this observation. However, this trend must be considered with caution, as only a few pairwise *post hoc* tests demonstrated statistically significant differences in the CPP for different flow rates. In addition, both HNR and CPP showed reasonable values compared with those reported in the literature (Verdolini et al., 1998; Patel et al., 2014b; Taylor and Thomson, 2022; Laukkanen et al., 2008). The same holds for Jitter and Shimmer, computed based on sound signal (Bielamowicz et al., 1996; Teixeira et al., 2013).

Finally, regarding the group of tissue elasticity parameters, the flow rate was found to have a significant influence on the ALR, which represents a characteristic increase in oscillation amplitude for an increasing flow rate (Patel et al., 2022).

In summary, while the flow rate influenced all evaluated oscillation characteristics (represented by the parameter groups), it appeared to have a stronger association with parameters that represent the amplitude of the vocal fold oscillation, such as AP, ASI, MADR, and ALR. The time-based or stiffness-related parameters F0, TP, PAI, and Stiff were only weak or even not at all, depending on the flow rate.

### 4.2 Impact of fiber tension on phonation parameters

Compared with the flow rate, the influence of fiber tension, represented by fiber stretching, on the parameter is more specific to certain parameter groups. One group (periodicity and symmetry) with no statistically significant differences was identified. However, the influence is stronger and more distinct in those parameters that demonstrate statistical significance, such as F0, Stiff, GGI, and CPP.

The most obvious impact was detected for stiffness, which is an alternative measure of elastic properties based on the glottal area waveform (Patel et al., 2022). Commonly, it is interpreted that higher values in stiffness resulting in a faster opening or closing of the glottis due to higher stiffness in the tissue, as the vocal folds oscillate with a higher frequency under applied pretension (Kist et al., 2021; Pelka et al., 2023; Titze and Martin, 1998; Roy, 2003). The two parameters regarding tissue elasticity indicate a statistically significant increase in the stiffness of the vocal folds with increasing fiber tension, reflecting physiological behavior (Zhang, 2015), where a higher stiffness is related to higher fundamental frequencies (Zhang, 2016). However, the pairwise tests revealed that the stretching levels from 10 to 20 mm were not significantly different, which appears to be a saturation effect due to excessive damping of fiber tension levels and suppression of vocal fold oscillations.

The model demonstrated fundamental frequencies in the range between 208.4 Hz and 380 Hz which reflects physiological conditions well. The physiological range of the fundamental frequency in men is approximately 100–220 Hz (Sundberg et al., 2005), whereas women have a slightly higher normal range of approximately 190–260 Hz (Cristina Oliveira et al., 2021; Jones and Garrett, 2016). In the model, the fundamental frequency had a maximum value of 380 Hz, which captured the frequency range of the vocally trained singers quite well. In professional female singers, the fundamental frequency can reach 1,500 Hz (Graham et al., 2016; Echternach et al., 2011). Furthermore, F0 increases with increasing fiber tension, which is also a characteristic of human phonation because the fundamental frequency is highly influenced by vocal fold tension during phonation (Tong and Sataloff, 2022; Jiang et al., 2000; Colton, 1988; Luizard et al., 2023). Based on the individual’s anatomical characteristics, this interaction results in characteristic voice pitches from person to person (Cavalcanti et al., 2021).

The periodicity and symmetry parameters (AP, TP, ASI, and PAI) were not significantly affected by an increase in symmetrically applied fiber tension. However, asymmetrical tension conditions may significantly influence periodicity and symmetry, as described by Xue et al. (2010) They found asymmetrical and non-periodic vocal fold oscillations in pathologies such as unilateral vocal fold paralysis, which highly affected the elastic characteristics of the vocal fold tissue.

The parameters describing the closing behavior of the vocal folds (GGI, ClQ, and MADR) demonstrated reasonable values. In most cases, the glottis does not fully close during the oscillation, starting with a high median GGI of 0.545 for the fiber stretching length of 0 mm, and decreasing to more physiological values for higher stretching length, which are statistically significant until the stretching length of 10 mm is reached. In previous *in vivo* and *ex vivo* studies the GGI has been reported to physiologically approach values close to 0 as this represents complete glottis closure (Semmler et al., 2021; Jakubaß et al., 2023; Peters et al., 2022; Semmler et al., 2023; Kniesburges et al., 2020; Graham et al., 2016; Zhang, 2019; Schlegel et al., 2019; Patel et al., 2012; Södersten et al., 1995). Many synthetic larynx models have faced limitations regarding glottis closure (Kniesburges et al., 2020; Zhang et al., 2006; Zhang et al., 2009). The model shows, that with higher elongation of the fibers, the glottis closure increases. Incomplete glottis closure, however, also takes place in physiological human phonation as it has been shown in females and children (Patel et al., 2012; Schneider and Bigenzahn, 2003; Cielo et al., 2019). The ClQ was not statistically significant; however, its values were reasonable compared to those found in previous studies (Semmler et al., 2023; Verdolini et al., 1998).

Although the overall variation of the MADR was statistically significant in the Kruskal-Wallis test with regard to the increase in fiber tension, the pairwise tests revealed only a significant increase in the MADR from 0 mm to 5 mm stretching length increase. With a further increase in tension, the range and median values decreased within the range of values found in the literature (Semmler et al., 2021).

Considering the acoustic parameters, an increase in fiber tension seems to increase the tonal content in the generated sound, as indicated by an increase in CPP and HNR, which is statistically significant for both parameters obtained from the Kruskal-Wallis test. Furthermore, the jitter also decreased with increasing tension, indicating an increase in the periodicity of the sound signal. The values of the acoustic parameters are comparable to those found in human phonation (Falk et al., 2021; Ferrand, 2002; Gaur et al., 2023; Heller Murray et al., 2022; Fraile and Godino-Llorente, 2014; Sørensen et al., 2016). Based on these results, it is clear that pathological or age-related changes in tissue elasticity play a major role in negatively affecting voice quality (Forero et al., 2016).

As mentioned earlier, the effect of saturation on high-tension levels was detected for various parameters: F0, GGI, MADR, Stiff, CPP, HNR, and Jitter. This effect appeared in the boxplots as constant or slightly decreasing ranges and median values for stretching levels of 10–20 mm. Furthermore, pairwise tests showed no significant differences between the value groups for the relevant tension levels, as listed in Table 5. The reason for this saturation is the potentially excessively high applied stretching levels in this range (10 mm–20 mm) which certainly exceed the physiological range of tension in collagen and elastin fibers.

### 4.3 Summary and conclusion

The model introduced by Tur et al. (2023) produces physiological oscillation patterns of the vocal folds and sound characteristics similar to those of human phonation. This study evaluated the model using parameters that commonly represent periodicity and symmetry, glottis closure, tissue elasticity, and acoustics commonly used in human phonation research. Therefore, the flow rate and tension in the fibers embedded in the synthetic vocal folds varied, which constituted the two laryngeal tuning factors controlling the fundamental frequency, loudness, and quality of the voice.

The data demonstrate the highly complex interaction between flow rate and longitudinal tension in the vocal folds and the large range of sound characteristics that can be adjusted by these two factors. The flow rate shows a large impact on nearly all phonatory characteristics, such as periodicity and symmetry, glottis closure, tissue elasticity, and acoustics. In contrast, the longitudinal tension within the vocal folds represented in the fiber tension model specifically influenced the fundamental frequency, glottis closing behavior, and production of tonal sound components in the sound signal. In particular, the acoustic parameters commonly used to analyze the sound quality produced during phonation increased, indicating a higher strength of tonal sound.

For pathological conditions, the flow rate is assumed to have larger potentials to compensate disorders in the neurological control and biomechanical conditions in the larynx as the flow rate is controlled by conditions in the lungs, the trachea and the muscles contraction of the thorax. However, this compensation strategy is limited as the air volume available for phonation is limited due to the individual lungs volume. Influencing laryngeal control mechanisms as pretension/stiffness in the larynx or vocal folds’ posturing for compensating pathological mechanisms in the larynx directly is, however, not simply to establish and requires surgical, conservative and logopedic therapy.

## Data Availability

The datasets presented in this article are not publicly available due to ongoing research. Requests to access the datasets should be directed to the corresponding author.
